# Sow Farrowing Early Warning and Supervision for Embedded Board Implementations

**DOI:** 10.3390/s23020727

**Published:** 2023-01-09

**Authors:** Jinxin Chen, Jie Zhou, Longshen Liu, Cuini Shu, Mingxia Shen, Wen Yao

**Affiliations:** 1 College of Engineering, Nanjing Agricultural University, Nanjing 210031, China; 2 College of Artificial Intelligence, Nanjing Agricultural University, Nanjing 210031, China; 3 College of Animal Science & Technology, Nanjing Agricultural University, Nanjing 210095, China

**Keywords:** sows in perinatal period, early warning and supervision of sow farrowing, lightweight deep learning, embedded development board, YOLOv5

## Abstract

Sow farrowing is an important part of pig breeding. The accurate and effective early warning of sow behaviors in farrowing helps breeders determine whether it is necessary to intervene with the farrowing process in a timely manner and is thus essential for increasing the survival rate of piglets and the profits of pig farms. For large pig farms, human resources and costs are important considerations in farrowing supervision. The existing method, which uses cloud computing-based deep learning to supervise sow farrowing, has a high equipment cost and requires uploading all data to a cloud data center, requiring a large network bandwidth. Thus, this paper proposes an approach for the early warning and supervision of farrowing behaviors based on the embedded artificial-intelligence computing platform (NVIDIA Jetson Nano). This lightweight deep learning method allows the rapid processing of sow farrowing video data at edge nodes, reducing the bandwidth requirement and ensuring data security in the network transmission. Experiments indicated that after the model was migrated to the Jetson Nano, its precision of sow postures and newborn piglets detection was 93.5%, with a recall rate of 92.2%, and the detection speed was increased by a factor larger than 8. The early warning of 18 approaching farrowing (5 h) sows were tested. The mean error of warning was 1.02 h.

## 1. Introduction

Early warnings and supervision of sow farrowing refer to alarms generated before the onset of sow farrowing to alert breeders so that they can detect and deal with related problems early on and alleviate problems such as difficult farrowing (dystocia), piglet suffocation, and excessively low temperatures during farrowing [1,2]. As manual inspection is time-consuming, labor-intensive, and highly subjective, automatic early warnings and supervision of approaching farrowing are important. They not only increase the average number of live born piglets per sow per year but also help piglets grow healthily and ensure the growth of the pig industry.

Sows start to show obvious nest-building behavior 24 h before the onset of farrowing with higher activity levels than normal and an increased frequency of posture changes. These behaviors can often be reliable indicators of farrowing [3,4]. Researchers worldwide have studied the nesting behavior of sows using various sensor devices, which can be mainly classified into contact and non-contact sensors. Oliviero et al. [5] installed a gravity sensor on the floor of a hog house and used a pressure-sensor mat and a photoelectric sensor to detect the duration of standing and the average frequency of the transition from standing to lying down for sows to predict the time of farrowing. An ear-tag accelerometer sensor designed by Traulsen et al. [6] can detect increases in the activity of sows and initiate an early warning device 6–8 h and then 1–2 h prior to the onset of farrowing. Manteuffel et al. [7] used a photoelectric sensor to qualitatively and quantitatively predict sow behavior in farrowing by using a dynamic linear model and cumulative sums. Küster et al. [8] analyzed the behavioral activities of sows via the dynamic background subtraction (DBS) and optical flow (OF) methods; this approach can provide warnings 4–6 h prior to farrowing on average. Okinda et al. [9] proposed a method for detecting piglets using an infrared depth image sensor, which can determine the time of sow farrowing, the exact birth time of each piglet, the birth order of piglets, the number of piglets born, etc. without interference, to detect asphyxiated piglets.

Methods of computer vision-assisted supervision have the advantages of persistence, non-invasiveness to animals [10], and strong resistance to interference. Although deep-learning algorithms [11,12,13] have developed rapidly, they require a large number of calculations and numerous parameters, and models can normally only run on high-performance workstations equipped with graphics processing units (GPUs). A computer-vision technology based on lightweight deep learning [14,15,16] has emerged owing to cost considerations. This paper proposes a lightweight deep learning-based approach for the early warning and supervision of sow behaviors preceding and during farrowing. A model was designed for detecting sow postures and piglets born via YOLOv5 algorithms and deployed on a Jetson Nano embedded development board. This migrated model can detect sows and newborn piglets of different sizes in complex scenarios such as a hog house. It allows early warning of approaching farrowing via posture detection and analysis of the frequency of postural transitions. Additionally, the model can generate alarms of approaching farrowing and determine the number of newborn piglets, thereby reducing the mortality of newborn piglets resulting from a lack of breeder support, improving sow management and productivity, and increasing the profits of pig-farming enterprises.

## 2. Materials and Methods

### 2.1. Animals, Housing, and Data Collection

#### 2.1.1. Animals and Housing

In this study, video data of sows were collected from two pig farms: Fengyuan Ecological Agriculture Co., Ltd. (Jingjiang City, Jiangsu Province, China) and the Zhengjie Pig Farm (Suyu District, Suqian City, Jiangsu Province, China). There was a total of 35 sows, 11 of which were studied at the Fengyuan Pig Farm during the period of 24 April 2017 to 31 May 2017. The other 24 were studied at the Zhengjie Pig Farm from 9 June to 15 June, 2020. Large white sows in the perinatal period and their piglets were selected as the test subjects for both farms. The sows were randomly placed in farrowing crates (2.2 m × 1.8 m) one week before farrowing. The stalls were equipped with food troughs, drinking bowls, piglet incubators, etc. The ambient temperature in the sow farrowing rooms was maintained between 20 and 28 °C, and the temperatures of the piglet incubators were controlled within a comfortable range in accordance with that of the farrowing room. The use of piglet incubators started 1 day before the expected farrowing date and stopped when the piglets were weaned.

#### 2.1.2. Data-Acquisition Equipment

**Figure 1:**
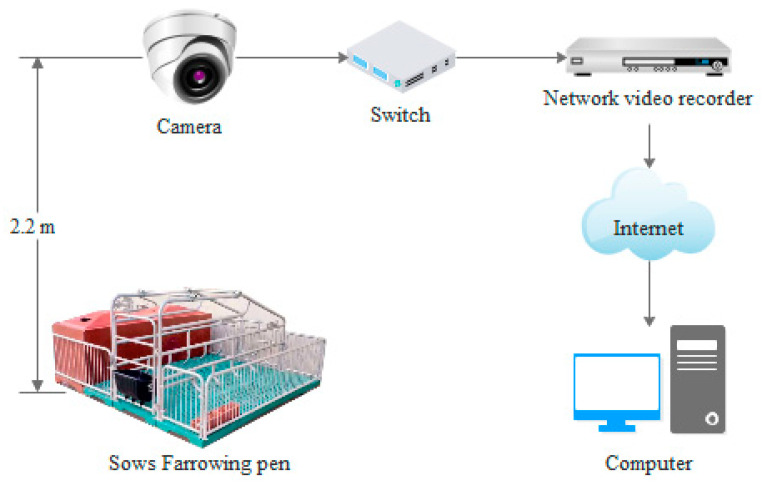
Sketch of video acquisition.

The video capture devices consisted of cameras, a network hard disk video recorder (NVR), a switch, and a local server. The cameras were installed in the farrowing rooms, and the switch, NVR, and local server were installed in a cabinet in an information service center adjacent to the farrowing rooms. Figure 1 presents a schematic of the camera devices. The same devices were used for the Fengyuan Pig Farm and the Zhengjie Pig Farm, except that the network camera models were slightly different. The camera models used at the Fengyuan Pig Farm and Zhengjie Pig Farm were DS-2CD3135F-I and DS-2CD3325-I, respectively, with resolutions of 2048 × 1536 pixels and 1920 × 1080 pixels, respectively. They had a focal length of 4 mm. They were located on the ceiling directly, 2.2 m above the farrowing crates, to ensure that the scope of supervision covered the entire crate. Each camera was connected to a network cable for power supply and data transmission, which was connected to a Power over Ethernet (PoE) switch (model: DS-3E0526P-E) through the crossbar of the fixed camera. The overhead videos of the pigs were recorded continuously for 24 h and then stored on the hard disk through the NVR (model: DS-8832N-R8) and imported to the computer.

#### 2.1.3. Data Preprocessing

The videos recorded 1 day before and after the sow farrowing were manually screened and then processed into image data using Python and OpenCV. The labeling software was used to manually annotate and enhance the data of sow postures and newborn piglets in the 12,450 images acquired. Data enhancement was performed via cropping, translation, rotation, mirroring, changing the brightness, adding noise, and cutout. A total of 32,541 images were acquired after the data were expanded. The dataset was divided into five categories, including four sow postures (lateral lying, sternal lying, standing, and sitting) [17,18] and the newborn piglets. The training set, validation set, and test set ratio was 7:1:2, and the dataset was produced in the PASCAL VOC standard format.

### 2.2. Object-Detection Algorithm (Computer-Vision Analysis)

**Figure 2:**
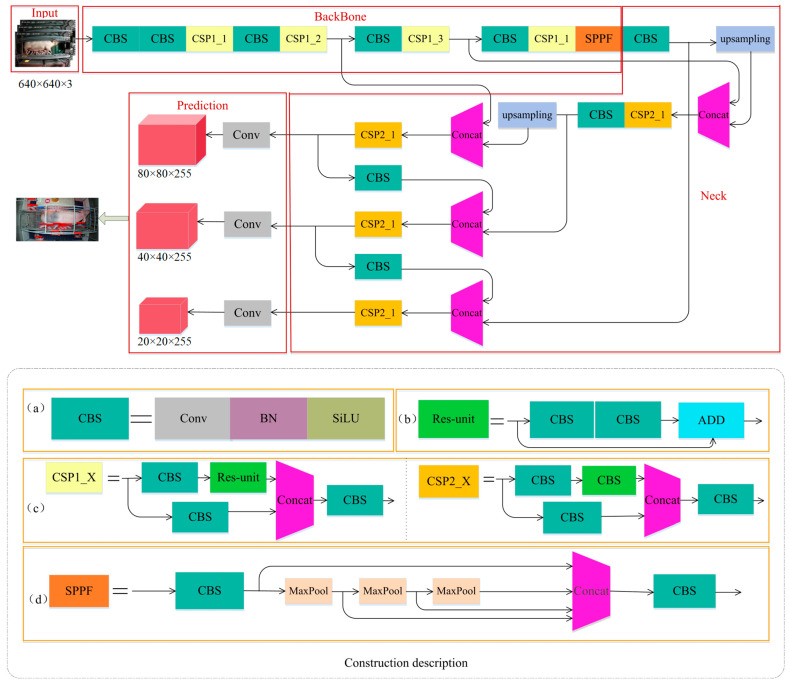
Network structure of the YOLOv5s algorithm (**a**) CBS module details, (**b**) Res-unit module details, (**c**) CSP1_X and CSP2_X module detail structure, and (**d**) SPPF module detail structure.

Using the same algorithm to detect sows and piglets concurrently is advantageous for lightweight deep learning. Hence, an algorithm suitable for the detection of targets of various sizes was designed. In this study, the YOLOv5s-6.0 network structure was used to build a model for detecting sow postures and newborn piglets. It is an anchor-based algorithm that is primarily composed of four parts: Input, BackBone, Neck, and Prediction. A detailed diagram of its structure is presented in Figure 2. The Input part is responsible for pig image input, the BackBone part extracts the image features of sows and piglets, and the Neck part fuses the features of pig images. Because sows and piglets differ significantly in size, three feature maps of different sizes are used in the Prediction part to detect small, medium-sized, and large objects.

The loss function of the model consists of three parts: Bounding Box Loss, Class Loss, and Object Loss. Bounding Box Loss uses the generalized IoU (GIoU) loss to calculate the loss for bounding-box regression, and Class Loss and Object Loss use the loss function of binary cross-entropy (BCEloss) to calculate these losses. GIoU loss and BCEloss are calculated using Equations (1) and (2), respectively.

(1) $$Loss_{\_GIOU}=1-(\frac{|A\cap B|}{|A\cup B|}-\frac{|C-(A\cup B)|}{\left|C\right|})$$

(2) $$Loss_{\_BCE}=-\sum_{n=1}^{5}y_{i}\log({\hat{y}}_{i})+(1-y_{i})\log(1-{\hat{y}}_{i})$$

Here, A and B represent the areas of the true box and prediction box, respectively; C represents the area of the minimum rectangle containing A and B; ${\hat{y}}_{i}$ represents the prediction value after the sigmoid() function is implemented, and $y_{i}$ represents the true value.

### 2.3. Model Optimization and Deployment

**Figure 3:**
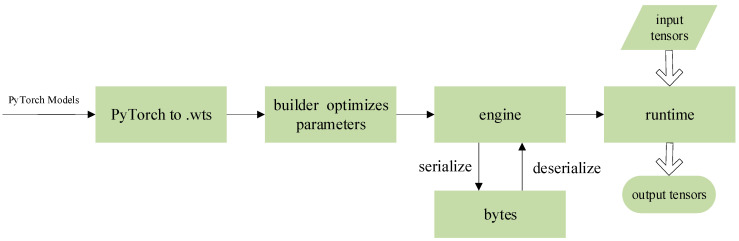
Workflow of TensorRT.

The algorithm for detecting sow postures and newborn piglets was deployed on the embedded artificial-intelligence (AI) computing platform of the Jetson Nano series launched by NVIDIA. TensorRT is a high-performance neural-network inference optimizer and runtime engine for Jetson Nano deployment. It was used in this study to optimize the proposed model based on the YOLOv5s algorithm. The conversion process of the model on the Jetson Nano embedded development board was pth→.wts→.engine, as shown in Figure 3. The optimized model ran on the embedded board with a higher throughput and lower latency and can avoid data security problems caused by data leakage during network transmission [19,20,21].

### 2.4. Model Evaluation Index

The performance of different algorithms was evaluated using indices such as the precision, recall rate, and detection speed. The size of the model and its detection speed indicated whether the algorithm was suitable for migration to embedded devices, and the precision and recall rate were used to measure the algorithm’s ability to detect all categories, including four sow postures (lateral lying, sternal lying, standing, and sitting) and the newborn piglets. The calculation formulas were as follows:

(3) $$P=\frac{TP}{TP+FP}$$

(4) $$R=\frac{TP}{TP+FN},$$

where *TP* represents the number of correct predictions for positive samples, *FP* represents the number of false predictions for positive samples, and *FN* represents the number of false predictions for negative samples.

## 3. Results and Discussion

### 3.1. Experimental Resources and Model Parameters

The model training environment was as follows: Ubuntu 18.04 operating system; Intel(R) Xeon(R) Gold 5118 @ 2.30 GHz CPU; NVIDIA Quadro P4000 GPU; 8 GB of video memory; 64 GB of memory; 2-TB hard disk; PyTorch 1.7.1 and Torchvision 0.8.2 deep-learning frameworks; CUDA version 10.1.

The model deployment environment was as follows: Ubuntu 16.04 operating system for ARM; 4-core ARM A57 @ 1.43 GHz processor (CPU); 128-core Maxwell architecture GPU; 4 GB of memory; JetPack 4.5; Cuda 10.2.89; Python 3.6, TensorRT 7.1, Opencv 4.1.1, and CMake 3.21.2 deep-learning environments.

The model parameters were as follows. (1) For the YOLOv5 training, the number of epochs was set as 300, the learning_rate was set as 0.0001, and the batch_size was set as 16. (2) For the TensorRT-optimized network, considering that a larger batch and higher precision require a longer processing time, the batch_size was set as 1, and fp16 precision was used.

### 3.2. Evaluation of Detection Performance

#### 3.2.1. Model Training Results and Analysis

**Figure 4:**
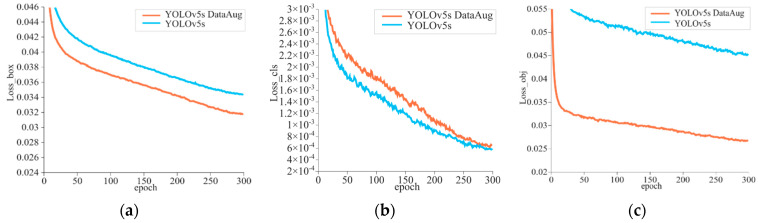
Loss curve of the training process. (**a**) Loss_box, (**b**) Loss_cls, (**c**) Loss_obj.

The loss function of the model indicates the degree of difference between the true value and the value predicted by the model. This difference decreased as the training process continues. The model performance is excellent when the loss value becomes stable. Figure 4 presents the training results of the model for detecting sow postures and newborn piglets. The loss curve exhibited an overall downward trend and tended to be fitted after 300 training epochs. Here, Figure 4a is the bounding box loss, Figure 4b is the class loss of target classification, and Figure 4c is the object confidence loss. Obviously, the data enhancement work makes these three losses converge more rapidly during the model training, and the final training is completed with less loss.

**Figure 5:**
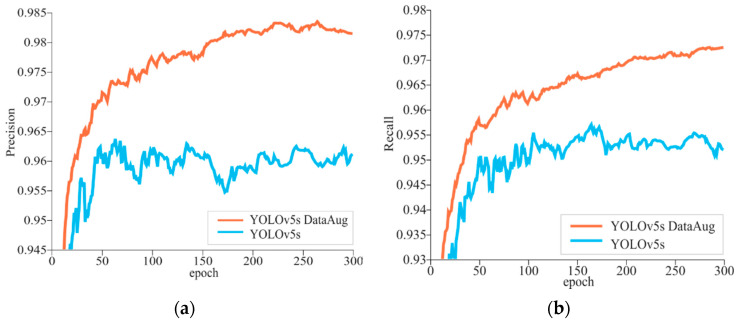
Precision and recall for the YOLOv5s detection model. (**a**) Precision, (**b**) Recall.

**Figure 6:**
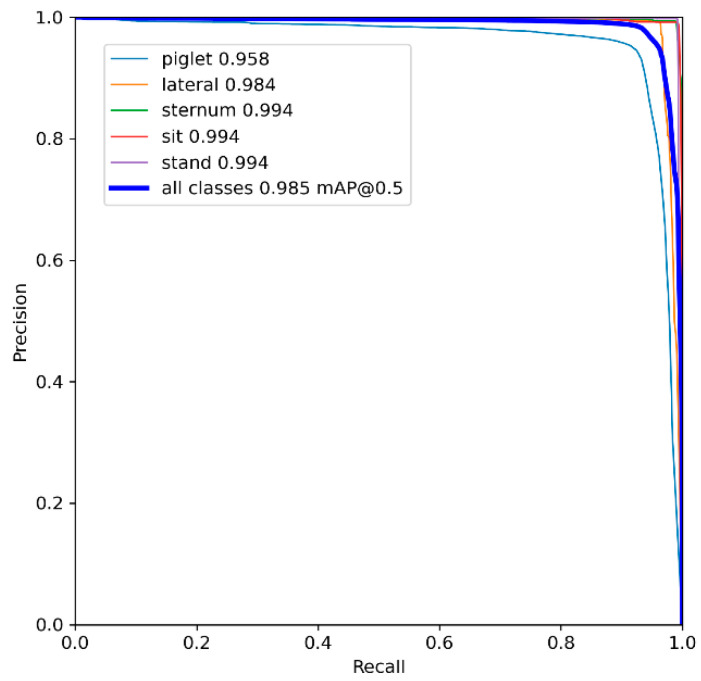
P-R curve for YOLOv5s_DataAug.

The change curves of the accuracy and recall of the model are shown in Figure 5. It can be seen that the precision rate in Figure 5a and the recall rate in Figure 5b show an overall increasing trend as the iteration period increases, but the YOLOv5s model trained by data augmentation consistently has higher precision and recall during the 300 epochs of training the YOLOv5s model. Figure 6 shows the precision–recall (P-R) curve of the model after data enhancement. The mean average precision (mAP) was used to evaluate the algorithm’s ability to detect all categories. The actual meaning in the graph is the area enclosed between the mean of the P-R curves of a total of five categories of sow stance and piglets and the coordinate axis. The model’s mAP reached 0.985 when the IoU threshold was 0.5.

In addition to the YOLOv5s algorithm, we evaluated the performance of the Anchor-free target detection algorithms YOLOX-nano and NanoDet-m—both of which support image detection, local video detection, and camera detection—for the same dataset. Table 1 presents the indices used to evaluate the model. A comprehensive analysis indicated that YOLOX-nano and NanoDet-m performed the detection slightly faster than YOLOv5s, whereas their precisions were lower, with numerous missed or false detections of newborn piglets. In contrast, the YOLOv5s algorithm was effective for the detection of different sizes, and the model’s average detection speeds for images, local videos, and cameras were 9.9, 9.8, and 8.1 ms, respectively, which is comparable to the detection speed of the other two Anchor-free target detection algorithms, and the data enhanced YOLOv5s model has the highest precision and recall rates, which are 0.982 and 0.973.

**Figure 7:**
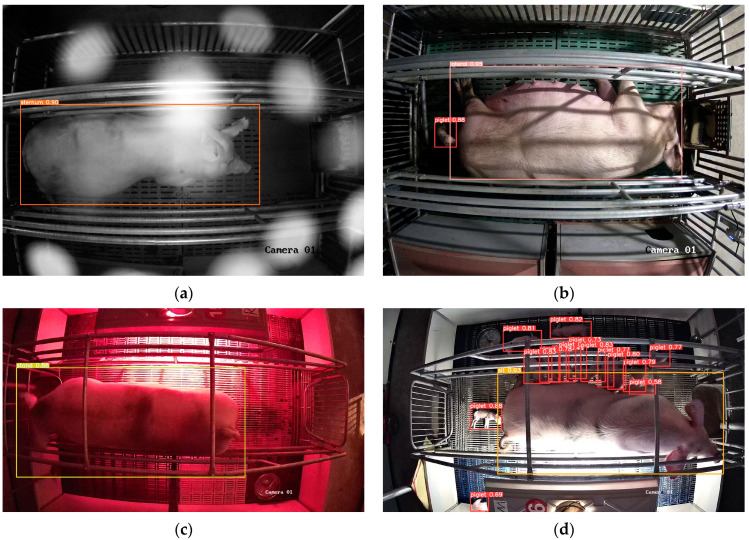
Complex scenarios image test effect. (**a**) Complex light, (**b**) First piglet born, (**c**) Different color of heat lamp, (**d**) Turn on heat lamp at night.

In order to test the generalization ability and anti-interference ability of the model in this paper, the sow numbered 24 at the Zhengjie Pig Farm, which was called “new sample”, was reserved when the model was trained. A total of 410 images containing different complex scenes were selected from the new sample to test the model. Figure 7 shows the results of sow posture and piglet target detection in four scenarios: complex light, the time of the first piglet’s birth, different colors of heat lamps, and turning on heat lamp at night. The missed and false detection of the model after testing are shown in Table 2. The results show that the missed and false detection of sow posture is mainly affected by the change of light (Figure 7a,d), the piglets are mainly affected by the heat lamp turning on, i.e., the piglets under strong light are difficult to identify (Figure 7d), and the scenarios of the time of the first piglet’s birth and different colors of heat lamps (Figure 7b,c) have little effect on the detection ability of the model.

#### 3.2.2. Model Deployment Test Results and Analysis

**Figure 8:**
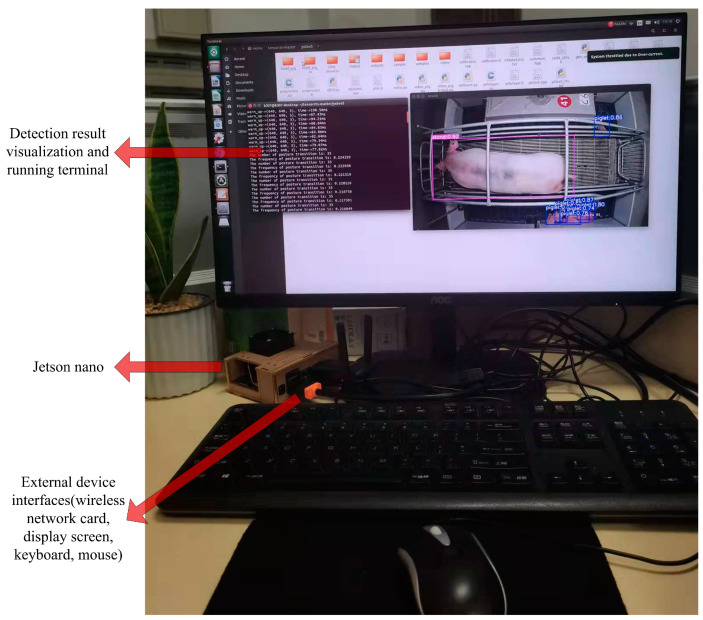
Jetson Nano running effect.

The wireless network card, monitor, keyboard, mouse, and other devices were connected to the Jetson Nano. A CF-938AC wireless network card with an M.2 interface was connect to a WiFi node to realize wireless data transmission. Decoding and neural-network inference were performed for the real-time video stream of the TensorRT optimized model for the detection of sow postures and newborn piglets. The model was then successfully deployed on the embedded development board, as shown in Figure 8.

**Figure 9:**
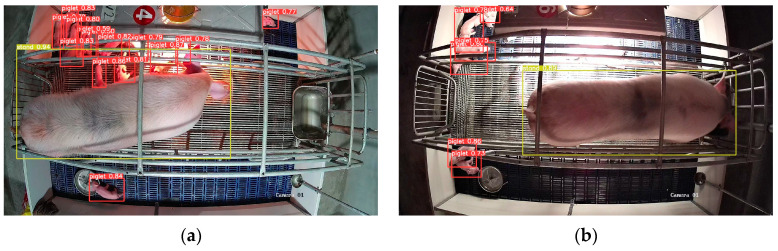

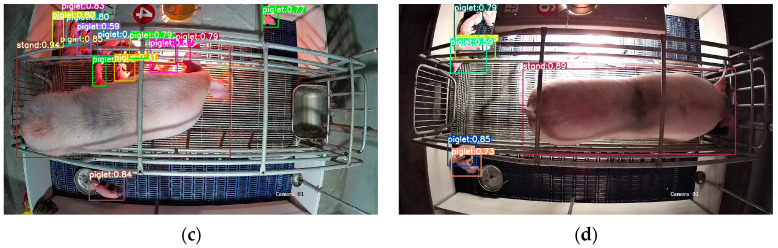
Test results before and after model optimization. (**a**) Test result on Quadro P4000 in daytime, (**b**) Test result on Quadro P4000 at night, (**c**) Test result on Jetson Nano in daytime, (**d**) Test result on Jetson Nano at night.

In this study, the detection results before and after the model optimization were tested for daytime and nighttime scenarios. As shown in Figure 9, Quadro P4000 was used in the test platform in the cases of Figure 9a,b, and Jetson Nano was used in the cases of Figure 9c,d. By comparing the model detection results between Figure 9a,c and Figure 9b,d for the same scenario, it can be found that the model accurately detected the sow postures and newborn piglets after being deployed to the embedded development board. Table 3 presents a detailed comparison of the test results. Although the model exhibited a minor loss of precision after optimization, its speed was increased by a factor larger than 8.

**Figure 10:**
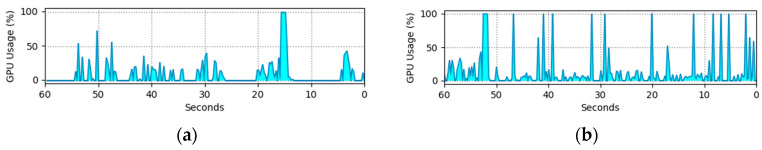
GPU utilization during the detection of images and video. (**a**) Image detection, (**b**) Video detection.

The GPU utilization rates on the embedded development board constrain the practical application capability of the model. Figure 10a,b present the GPU utilization rates when the model detected targets in the form of images and videos on the embedded development board. As decoding was required for processing the video streams, the GPU utilization rate during the detection of videos was higher than that during the detection of images, although this did not affect the performance of the model. The two test results show that the model in this paper can be applied to different production scenarios.

### 3.3. Implementation of Sow Farrowing Early Warning and Supervision

According to the posture sequences of the sows at different moments output by the model, the sow posture transition frequency from 48 h before farrowing to 24 h after farrowing was calculated as follows:

(5) $$f=\frac{n}{T}$$

where *f* represents the sow posture transition frequency, *n* represents the number of posture transitions, and *T* represents time (unit h). The values were continuously updated during the detection process.

**Figure 11:**
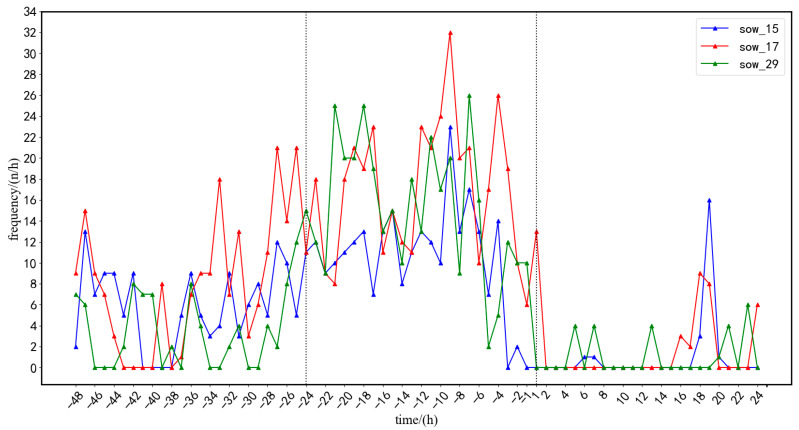
Visualization of the frequency of sow posture change (three sows are taken for example).

**Figure 12:**
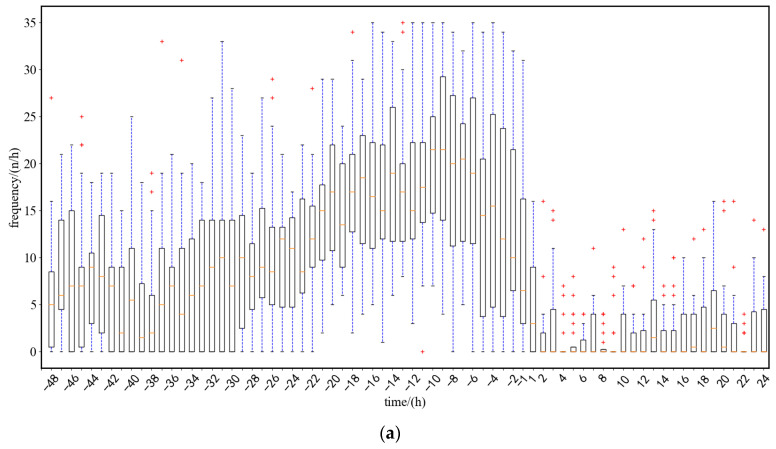

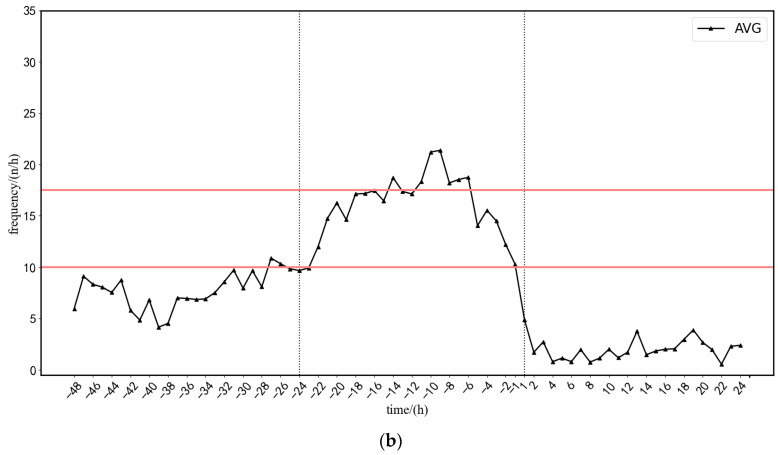
Range and mean of sow per hour posture change frequency (results of 22 sows statistics). (**a**) Frequency range of sow posture change per hour. (**b**) Mean frequency of sow posture change.

As shown in Figure 11, three sows were selected as examples to visually analyze the posture transition frequency, which changed in a consistent direction across sows. The data of 22 sows from the experimental sample were tested and analyzed, and the range of change in the average posture change rate of sows from 48 h before farrowing to 24 h after farrowing is shown in Figure 12a, and the average posture change frequency of sows calculated on this basis is shown in Figure 12b. Based on the results presented in Figure 12, the sow farrowing behaviors were roughly divided into three stages: (1) 48 to 24 h before farrowing, during which the sows exhibited normal activities; (2) 24 to −1 h before farrowing, during which the posture transition frequency significantly increased and then decreased; and (3) 1 to 24 h after farrowing, during which the frequency approached 0 and then increased slightly.

Considering the aforementioned patterns, the early-warning strategies used in this study can be summarized as follows: (1) the early warning was sent when the sows’ posture transition frequency exceeded the upper threshold of 17.5 times/h (2) and when it fell below the lower threshold of 10 times/h; (3) for minimizing the impact of daily living habits (such as eating, drinking, and resting) of the sows on the warning of approaching farrowing, the upper or lower threshold had to be exceeded for more than 5 h. Tests on the 22 sows used as the experimental samples revealed that early warnings could be sent 5 h prior to the onset of farrowing, with an average error of 1.02 h between the early-warning time and the actual farrowing time.

Sows’ farrowing behaviors were supervised according to the result of newborn piglet detection to implement early warning of farrowing and determine the number of piglets. The following formulas were used:

(6) $$\mathrm{Alarm}=\{\begin{array}{c} 1,\;T_{start}\neq Null\\ 0,\;T_{start}=Null \end{array}$$

(7) $$D=T_{end}-T_{\mathrm{start}}$$

where Alarm denotes the start of farrowing; *D* represents the farrowing duration, i.e., the time gap from the start to the end of farrowing; *T_start_* represents the time the first piglet was born, at which the farrowing started; and *T_end_* represents the time the last piglet was born, at which the farrowing ended.

**Figure 13:**
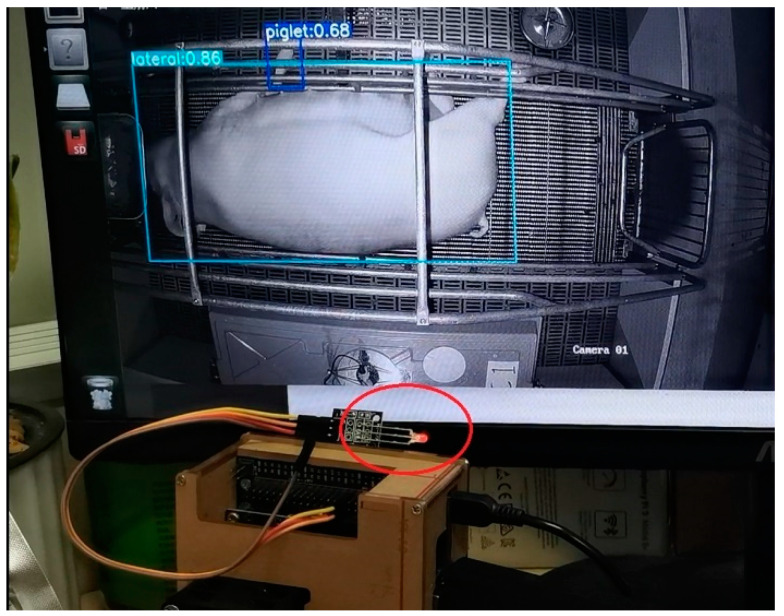
Sow farrowing alarm.

After the first newborn piglet was detected, the alarm for sow farrowing was triggered, prompting “Delivery Begin! Start time: ×××”. Additionally, the flashing of the light-emitting diode (LED) was controlled by varying the levels output by the General Purpose Input/Output (GPIO) pin. The breeder can quickly locate the farrowing sow by checking the flashing LED light and judge whether manual intervention is needed. The detection time of each image using the model on the embedded development board was 67.2–80.3 ms. When the detection speed was excessively high, piglets tended to be falsely detected, and the alarms were generated earlier than necessary. Hence, to reduce the number of false alarms and realize real-time detection, the approach of “three consecutive detections” was adopted before alarm generation; i.e., only when the newborn piglets were detected three times consecutively was farrowing judged to have occurred, as shown in Figure 13. Videos of the farrowing processes of 22 sows were selected for testing. The average number of false alarms was 9.55 when the “single detection” approach was adopted and 1.59 when the “three consecutive detections” approach was adopted, indicating a significant reduction in the number of false alarms.

**Figure 14:**
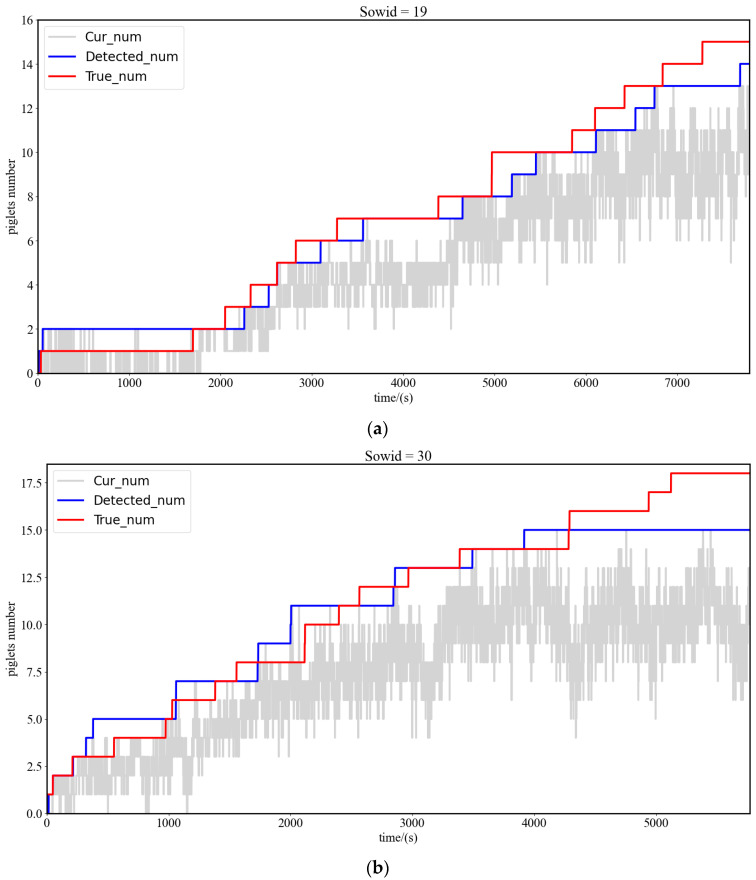
Test results for the number of piglets (two sows are taken for example). (**a**) Test results for the number of piglets (Sowid = 19), (**b**) Test results for the number of piglets (Sowid = 30).

Each time a tag-box for a piglet was detected, the number of piglets was increased by 1, and the duration of farrowing was recorded until the process ended and the number of piglets was determined. Figure 14 shows the number of piglets detected, with the 19th and 30th sows taken as examples. Cur_num represents the number of piglets within the supervision range with the maximum value being the piglet number. The number of piglets detected was close to the true value. The errors were primarily due to factors such as the adhesion of piglets, piglets being covered, and local parts of sows (such as ears, tails, legs) being falsely detected as piglets. The formula to measure the accuracy of piglet target detection during sow farrowing is as follows.

(8) $$CA=\frac{\sum_{i=0}^{n}Cur\_num_{i}}{\sum_{i=0}^{n}True\_num_{i}}$$

(9) $$DA=\frac{\sum_{i=0}^{n}Detected\_num_{i}}{\sum_{i=0}^{n}True\_num_{i}}$$

In the above equation, CA (Current Number Accuracy) is the average accuracy of the current number of piglets during sow farrowing, and DA (Detected Number Accuracy) is the average accuracy of piglet detection during sow farrowing. The overall average accuracy of the current number was 63.2% and the overall average accuracy of the detected number was 92.9% for the piglet detection during farrowing of 22 sows in the experimental sample, and the specific results of piglet detection per pen are shown in Table 4.

In real-world applications, cameras are installed on the embedded development board to detect newborn piglets in real time, and early warnings and alarms of approaching farrowing are sent according to the results of the detection of sow postures and piglets to communicate sows’ farrowing behaviors to breeders in a timely manner and increase the survival rate of piglets.

## 4. Conclusions

Early warnings of approaching farrowing and supervision of sows’ farrowing behaviors in the perinatal period were implemented using an embedded development board. The following conclusions are drawn.

(1) Video data of 35 sows before and after farrowing were collected and preprocessed to construct a dataset containing four types of sow postures as well as newborn piglets, and the model employed for the detection of sow postures and newborn piglets was trained and tested using Quadro P4000. An analysis of the evaluation indices of different algorithms revealed that YOLOv5 algorithms were effective for detecting sows and piglets of different sizes.
(2) An inference engine that supports TensorRT was generated for the model for the detection of sow postures and newborn piglets. The accelerated model was deployed to the embedded development board, after which it ran with a higher throughput and lower latency. The precision of the model after migration to Jetson Nano reached 93.5%, with a recall rate of 92.2%, and the detection speed was increased by a factor larger than 8.
(3) According to the changes in the frequency of sow posture transitions output by the model, early warnings were sent 5 h before the onset of farrowing, with an average error of 1.02 h between the warning time and the actual farrowing time. Alarms for sow farrowing were sent using the approach of “three consecutive detections” for the first newborn piglet, and the piglet number was determined according to the number of newborn piglets detected.

We investigated how to automatically predict and detect the duration of sow farrowing via early warning of approaching farrowing and supervision of farrowing behaviors based on the embedded development board. Compared with the method of Steffen Küster et al. [8], the error time of sow farrowing warning obtained by the method proposed in this paper is reduced by nearly 2 h and is deployed on an embedded development board. It has the advantages of a low cost, a low latency, a high efficiency, and easy implementation and can accelerate the transition to intelligent pig breeding.

## Figures and Tables

**Table 1:** Evaluation indices of different algorithms for sow posture and piglet object detection.

Model	Precision	Recall	Speed_Image	Speed_Video	Speed_Camera
YOLOX-nano	0.929	0.779	20.4 ms	4.8 ms	7.3 ms
NanoDet-m	0.967	0.833	8.0 ms	4.4 ms	4.2 ms
YOLOv5s	0.961	0.952	-	-	-
YOLOv5s_DataAug	0.982	0.973	9.9 ms	9.8 ms	8.1 ms

**Table 2:** Test situation of YOLOv5s model in complex scenarios.

Scenarios	Missed Detection Rate for Sow Posture	False Detection Rate for Sow Posture	Number of Missed Detections of Piglets	Number of False Detections of Piglets
Complex light	6.33%	15.19%	0	11
First piglet born	-	-	2	5
Different color of heat lamp	0	1.2%	7	1
Turn on heat lamp at night	3.36%	7.56%	15	10

**Table 3:** Model test effect comparison.

Model Format	Platform	Speed_Image	Speed_Video	Precision	Recall
.pth	Quadro P4000	9.9 ms	9.8 ms	0.982	0.973
.pth	Jetson Nano	583.4 ms	591.4 ms	0.982	0.973
.engine	Jetson Nano	67.2 ms	80.3 ms	0.935	0.922

**Table 4:** Results of the number of piglets detected per pen.

Sow ID	CA	DA	Sow ID	CA	DA	Sow ID	CA	DA
03	44.9%	95.0%	15	61.7%	84.4%	25	58.8%	99.3%
04	50.7%	91.7%	16	42.3%	90.3%	26	56.3%	83.6%
05	58.1%	94.1%	17	52.5%	89.9%	27	58.6%	87.1%
06	75.2%	95.9%	18	57.9%	92.1%	28	66.1%	99.4%
07	52.2%	96.4%	19	69.0%	95.2%	29	82.3%	88.0%
08	66.6%	96.8%	20	93.6%	98.3%	30	66.2%	98.4%
09	65.7%	94.3%	21	73.9%	99.6%			
10	66.2%	81.2%	22	71.0%	93.1%			

## Data Availability

Not applicable.
